# Vocal fold paralysis in subacute thyroiditis

**DOI:** 10.1016/S1808-8694(15)31141-1

**Published:** 2015-10-20

**Authors:** Rogério Aparecido Dedivitis, Leonardo Santos Coelho

**Affiliations:** 1 Medical doctor, doctor in medicine graduated from the Post-Graduate Otorhinolaryngolocial and Head & Neck Surgery Course at UNIFESP - Paulista Medical School; 2 Medical student of the Medical School of the Santos Metropolitan University Health Sciences College

**Keywords:** dysphonia, vocal fold paralysis, thyroiditis, thyroid

## INTRODUCTION

Vocal fold paralysis is associated with involvement of the vagus nerve or the recurrent laryngeal nerve between the jugular foramen and the entry point into the larynx. Malignancies of the thyroid are a frequent cause, but vocal fold paralysis is also found in certain benign diseases. In a series of 1,200 cases, the incidence of the association between paralysis and benign thyroid disease was 0.69%.^1^

In this paper we report a case of vocal fold paralysis associated with a benign thyroid condition.

## CASE REPORT

A female patient aged 43 years presented with a painful cervical nodule in the anterior inferior portion of the neck, and persistent dysphonia with a sudden onset five days ago. During the previous week she had had a common cold for which she used a non-steroidal anti-inflammatory drug. The clinical examination revealed a hard, painful nodule, mobile upon swallowing, measuring about 5.0 × 3.5 cm, located in the right thyroid lobe. Laryngoscopy showed right vocal fold paralysis in the paramedian position, and a fusiform slit between vocal folds on phonation. Prednisone (40 mg/day) was given during five days. Ultrasound revealed a nodule measuring 6.2 × 3.9 × 3.4 cm in the right thyroid lobe. The patient was seen again after seven days; the nodule was still palpable, but painless. The patient reported that voice quality had improved suddenly after three days of medication (prednisone). Laryngoscopy showed normal vocal fold mobility. Laboratory exams included elevated free thyroxin (2.1 mg/dl), lowered thyrotropin (0.105) and normal antithyroperoxidase and antithyroglobulin antibodies. Cytopathology of material obtained by fine needle aspiration revealed nodular goiter with cystic degeneration. After three weeks, thyroid hormone levels became normal. Hemithyroidectomy was indicated due to the size of the nodule, which displaced the trachea. Frozen section examination and definitive histopathology confirmed the diagnosis of adenomatous goiter. Follow-up laryngoscopy showed continued normal vocal fold mobility. Thyroid hormone levels one month after surgery were within normal limits.

## DISCUSSION

There are many causes of vocal fold paralysis, classified according to the site of the lesion or the etiology. This includes trauma, neoplasms, and mechanical, central nervous system, toxic, metabolic, inflammatory or idiopathic causes. A frequent cause is trauma resulting from thyroidectomy or thoracic/mediastinal surgery. There may be an association with certain tumors, such as those of the lungs and the thyroid gland.^2^

The paralysis mechanism in this situation remains unclear; theories include compression caused by a large goiter, calcification or inflammation, and pressure on the nerve against the trachea. In the acute inflammatory phase there could be edema or thrombosis of recurrent laryngeal nerve blood vessels. In the chronic phase, the possibility is perineural fibrosis.^3^ Paralysis may be a complication of subacute thyroiditis, and may persist even after clinical and laboratory recovery.^4^

Corticosteroids are the treatment of choice and should be started immediately. Spontaneous regression has been occasionally reported. The prognosis of vocal fold paralysis is somber if there is no improvement after nine months.^5^

The association between vocal fold paralysis and thyroiditis is rare. Vocal fold paralysis does not necessarily mean the presence of malignancy.
